# Gait Variability before Surgery and at Discharge in Patients Who Undergo Total Knee Arthroplasty: A Cohort Study

**DOI:** 10.1371/journal.pone.0117683

**Published:** 2015-01-24

**Authors:** Yoshinori Hiyama, Tsuyoshi Asai, Osamu Wada, Hideto Maruno, Shingo Nitta, Kiyonori Mizuno, Yasunobu Iwasaki, Shuichi Okada

**Affiliations:** 1 Anshin Hospital, Kobe, Hyogo, Japan; 2 Graduate school of Human Development and Environment, Kobe University, Kobe, Hyogo, Japan; 3 Department of Physical Therapy, Faculty of Rehabilitation, Kobegakuin University, Kobe, Hyogo, Japan; Van Andel Institute, UNITED STATES

## Abstract

This study aimed to determine gait ability at hospital discharge in patients undergoing total knee arthroplasty (TKA) as an indicator of the risk of falling. Fifty-seven patients undergoing primary TKA for knee osteoarthritis participated in this study. Gait variability measured with accelerometers and physical function including knee range of motion (ROM), quadriceps strength, walking speed, and the Timed Up and Go (TUG) test were evaluated preoperatively and at discharge from the hospital (1 month before and 5 days after surgery). All patients were discharged directly home at 5 days after surgery. Knee flexion of ROM, quadriceps strength, walking speed, and the TUG test results were significantly worse at hospital discharge than preoperatively (p < 0.001). However, gait variability was not significantly different before and after TKA. This result indicated that patients following TKA surgery could walk at hospital discharge as stably as preoperatively regardless of the decrease in physical function, including knee ROM, quadriceps strength, and gait speed after surgery.

## Introduction

Total knee arthroplasty (TKA) is the most common surgical intervention for end-stage knee osteoarthritis (OA) and offers patients pain relief, functional recovery, and improved quality of life [1, 2]. An estimated 70,000 TKA procedures are performed yearly in Japan, and the number increases annually. Early initiation of rehabilitation treatment after a TKA, such as interventions to increase joint range of motion (ROM) and quadriceps strength, is becoming popular and is considered important for obtaining the maximum benefit from knee arthroplasty [3–5]. Moreover, the early initiation of rehabilitation is beneficial for early hospital discharge [3–5]. Early hospital discharge is expected to lower the risk of hospital-acquired infection [6] and allows patients to quickly return to their daily life activities after the surgery. However, few studies have reported on patient outcomes such as gait ability at hospital discharge, and risk assessment such as the risk of falling at hospital discharge is essential so that patients can return home quickly and safely.

Falls are among the most common cause of injury, and knee OA is an important risk factor for falls [7–9] with more than 50% of people with knee OA reporting falls in the past year [10, 11]. Additionally, patients who undergo TKA are at increased risk of falling [12, 13]; however, no study has assessed the risk of falling at hospital discharge in patients who undergo TKA. Previous studies have identified many contributing factors for falls in older people, such as muscle weakness, impaired proprioception, balance impairment, and pain [14–16]. Especially, stride time variability is strongly correlated with the risk of falling in older people [17–22]. Hausdorff et al. measured the stride time variability in 52 community-living older people and found that the stride time variability in people who subsequently fell was greater than that in people who did not experience a fall during the follow-up period [17]. Additionally, they reported that the stride time variability predicted falls but other measures including strength, balance, and walking speed did not discriminate future fallers from nonfallers [17]. Therefore, stride time variability is becoming a relevant marker of gait stability and a useful predictor of falls both in research and clinically. Thus, the objective of this study was to investigate the stride time variability as a measure of gait ability at hospital discharge in patients undergoing TKA in order to assess the risk of falling.

## Methods

This was a prospective cohort study. The ethics committee of Anshin Hospital approved all procedures performed in this study before testing, and all patients provided written informed consent before participating. The target population consisted of a convenience sample of patients undergoing primary TKA for knee OA at our hospital; 57 patients who underwent primary unilateral TKA for knee OA were recruited. Inclusion criteria were as follows: (i) completion of primary TKA for knee OA staged as grade 3 or 4 based on the Kellgren and Lawrence radiographic grading system [23] (radiographic evaluation was performed by an independent experienced assessor); (ii) no demonstrable symptoms in the hip, ankle, and contralateral knee while walking; and (iii) being able to walk without a cane to eliminate the influence of the cane on gait ability; and (iv) provision of informed consent to participate in this study. Exclusion criteria were as follows: (i) postoperative complications such as deep vein thrombosis (DVT) and fall; (ii) neurological conditions such as Parkinson disease or other previous history affecting gait ability; (iii) limited walking with or without a cane postoperatively; and (iv) diagnosis of rheumatoid arthritis.

### Procedures

After informed consent was obtained, all patients started about 8 weeks (once every other week; 4 visits) of outpatient prehabilitation. The prehabilitation program included exercises such as ankle pump, hip adduction, quadriceps setting, and active assisted ROM exercises to improve knee ROM and muscle strength. Additionally, all patients were evaluated 1 month preoperatively and on the day of hospital discharge. The evaluation included measurements of gait variability and physical function. Regular inpatient physical therapy was started on postoperative day 1 and followed a standard protocol of ambulation exercises and activities of daily living exercises. Ambulation exercises began using a walker. After achieving independent ambulation with a walker, ambulation exercises using a cane were introduced. Additionally, after achieving independent ambulation with a cane, patients were permitted to practice descending and ascending stairs. The mobility-related goal at discharge was independent and safe ambulation and the ability to go up and down the stairs while holding the railing. Physical therapists and nurses defined safe ambulation as steady walking for an 80-m distance.

### Assessment of gait variability

Previously described methods were used to measure stride time variability [17–19,]. Briefly, patients were instructed to walk on a smooth, horizontal, 16-m walkway with a 3-m space before each end of the walkway for acceleration and deceleration at their normal speed. To measure stride time variability, Hausdorff et al. [19] instructed subjects to walk for up to 6 min; however, in this study, we instructed patients to walk along a 16-m walkway [24] to prevent them from increasing pain. The time taken to complete the 10-m distance walk was recorded to a hundredth of a second using a stopwatch and the results of 2 trials were averaged to obtain the gait speed. To control for the potential confounding influence of shoes, all patients wore the same kind of shoes.

A triaxial accelerometer (MVP-RF8-HC; Microstone Co., Nagano, Japan) was attached over the shank of the involved limb (3 cm proximal to the lateral malleolus) [25] using a Velcro belt. This site was selected to identify the initial contact time (heel strike) of each stride throughout walking and to minimize soft tissue oscillations during the impact without restricting movement. Before measurement, the accelerometer was attached over the shank of the involved limb and statically calibrated against gravity. All accelerations were sampled at 500 Hz, and all acceleration signals were synchronized. After analog-to-digital conversion, signals were collected in a data logger (MVP-RF8-S; Microstone Co.) and immediately transferred to a laptop computer through a Bluetooth personal area network. Signal processing was performed using Matlab Release R2013a (MathWorks, Natick, MA, USA). All acceleration data were low-pass filtered using a dual-pass zero-lag Butterworth filter with a cut-off frequency set at 20 Hz. All analyses were performed using data from the middle 10 strides during the steady walk in each test. Subsequently, the recorded signal was automatically analyzed to determine the heel strike of each stride throughout the walk, and hence, the stride time (the time from one heel strike to the next heel strike of the same foot) time series [26]. Stride time variability, the magnitude of the stride-to-stride fluctuations in the gait cycle duration, was calculated by determining the standard deviation (SD) and coefficient of variation (CV; [SD/mean] × 100%) of each patient’s stride time [18, 27]. The smaller the CV value, the better the postural stability was during gait. The results of the 2 trials were averaged to obtain the stride-time SD and CV.

### Physical function

Outcome measures of physical function included knee ROM, pain assessment, quadriceps strength, and the Timed Up and Go (TUG) test.

Knee ROM was measured using a standard 2-arm plastic goniometer, with the axis of the goniometer placed over the lateral epicondyle of the femur, the proximal arm aligned with the greater trochanter of the femur, and the distal arm aligned with the lateral malleolus of the ankle. Passive knee flexion and extension ROM were performed in the supine position with full passive knee ROM.

In the TUG test, patients were instructed to stand up from a 40-cm-high chair with no seat arms, walk a 3-m distance at a normal pace, turn, walk back to the chair, and sit down [28]. The time was recorded to one-hundredth of a second using a stopwatch, and the results of 2 trials were averaged to obtain the TUG test score. This test is reliable and valid to assess group change among inpatients undergoing orthopedic rehabilitation [29].

A numeric rating scale (NRS) was used to quantify knee pain during the TUG test. Immediately after the 2 tests, patients were asked to verbally rate the pain in and around the knee joint on a scale from 0 to 10, with 0 representing no pain and 10 representing the worst pain imaginable. The rating assigned during the attempt that produced the greatest force was used for analysis. The NRS is valid, reliable, and appropriate for use in clinical practice and has good sensitivity [30].

Quadriceps strength was measured as the peak isometric knee extension torque (Newton-m/kg) using a hand-held dynamometer (μTas F1; ANIMA, Chofu, Japan). Patients sat, with their hands on their laps, on a chair designed to stabilize the body and minimize synkinetic movements. The knee joint was held at an angle of 90°, and a belt was used to restrain the dynamometer to enhance the reliability of isometric leg muscle strength measurements [31]. The patients were asked to extend their knee (by using the command “kick as hard as possible”) for 3 s. During each test, consistent verbal encouragement was provided to the patients. The peak torque was estimated as the product of force and the distance between the attachment of the dynamometer (at the lateral malleolus) and the center of rotation of the knee joint. Two attempts of maximal contraction were performed, and the greater of the 2 measurements was recorded and normalized to body weight. The quadriceps strength of both the surgical and nonsurgical limbs was measured.

### Statistical analysis

Pre-protocol analysis was conducted after excluding patients who dropped out of the study. Results are reported as mean ± SD and median (range). The normality of the parameter’ distribution was verified with the Kolmogorov-Smirnov test. First, the paired *t*-test or the Wilcoxon signed-rank test was used to compare physical function before and after surgery. Additionally, the paired *t*-test or the Wilcoxon signed-rank test was also used to compare stride time SD and CV to examine the changes of gait fluctuation before and after surgery in patients with and without a need for a cane, respectively. A *p*-value < 0.05 (two-tailed) was considered statistically significant. Statistical analysis was performed using SPSS for Windows version 22.0 (IBM, Tokyo, Japan).

## Results

This study included 57 patients who had undergone TKA. Of the 57 patients initially enrolled in the study, 13 patients (22.8%) dropped out because of DVT and 1 patient (1.8%) dropped out because of health conditions. All patients were discharged directly home at 5 days after surgery, and all of them completed the assessment at the day of discharge.

The Kolmogorov-Smirnov test showed that the values of physical function followed a normal distribution but stride time SD and CV did not. Therefore, we used the paired *t*-test to compare physical function before and after surgery, and used the Wilcoxon signed-rank test to compare stride time SD and CV before and after surgery. Of the resulting 43 patients (Table 1), at hospital discharge, 20 patients could walk without a cane and 23 patients needed a cane during walking. Table 2 shows the results of preoperative and postoperative physical function. Knee ROM and quadriceps strength in the involved limb were significantly worse postoperatively than preoperatively (*p* < 0.001) (Table 2). Additionally, walking speed was significantly slower at hospital discharge than preoperatively (*p* < 0.001; Table 2). Table 3 shows the stride time variability before and after surgery. The Wilcoxon signed-rank test revealed no significant difference in stride time SD and CV between preoperative and postoperative values in both patients requiring and not requiring a cane during walking (Table 3).

**Table 1:** Characteristics of patients in this study.

	**Patients (n = 43)**	**Range**
Age (years), mean ± SD	72.0 ± 6.6	56–81
Women, n (%)	35 (81.4)	
Body mass index (kg/m^2^), mean ± SD	25.9 ± 3.3	19.1–33.4
Grade of osteoarthritis in the involved limb, n (%)		
Grade 3	3 (7.0)	
Grade 4	40 (93.0)	
Grade of osteoarthritis in the uninvolved limb, n (%)		
Grade 1	1 (2.3)	
Grade 2	9 (20.9)	
Grade 3	11 (25.6)	
Grade 4	22 (51.2)	

SD: standard deviation

**Table 2:** Preoperative and postoperative physical function.

	**Preoperative status**	**Postoperative status**	***p-value***
	**Mean ± SD**	**Mean ± SD**	
Knee range of motion in the involved limb			
Flexion (degree)	125.8 ± 13.3	93.8 ± 10.5	<0.001
Extension (degree)	-9.1 ± 6.1	-2.4 ± 3.8	<0.001
Knee range of motion in the uninvolved limb			
Flexion (degree)	134.5 ± 10.6	-	
Extension (degree)	-5.0 ± 5.2	-	
Quadriceps strength (Newton-m/kg)			
Involved limb	1.01 ± 0.39	0.30 ± 0.24	<0.001
Uninvolved limb	1.17 ± 0.43	-	
Walking speed during 10-m walking (m/s)	1.0 ± 0.2	0.7 ± 0.2	<0.001
Timed Up and Go (TUG) test (s)	10.0 ± 2.6	15.2 ± 4.1	<0.001
Pain during the 10-m walking test	2.6 ± 2.3	3.2 ± 2.4	0.12
Pain during TUG test	2.3 ± 2.3	3.5 ± 2.4	0.008

*p*-values are given for the significant difference by using the paired *t*-test.

**Table 3:** Walking speed and stride time variability.

	**Patients walking without a cane (n = 20)**		***p*-value**	**Patients walking with a cane (n = 23)**		***p*-value**
	**Preoperative**	**Postoperative**		**Preoperative**	**Postoperative**	
Stride time SD (ms)						
Mean ± SD	152.1 ± 72.6	176.3 ± 62.4	0.21	148.0 ± 78.8	195.6 ±89.4	0.31
Median (range)	150.0 (15.0–314.0)	163.6 (21.0–417.6)		150.0 (21.0–395.2)	184.4 (21.2–418.5)	
Stride time CV (%)						
Mean ± SD	13.4 ± 14.0	14.7 ± 13.2	0.33	13.7 ± 14.7	15.0 ± 13.1	0.95
Median (range)	8.9 (2.1–42.2)	10.4 (2.1–37.9)		8.6 (2.2–40.1)	8.7 (2.5–41.7)	

*p*-values are given for the significant difference by the Wilcoxon signed-rank test.

SD: standard deviation; CV: coefficient of variation

## Discussion

The present study found that stride time variability showed no significant pre- to postoperative change. On the other hand, physical function including knee ROM, quadriceps strength, and gait speed decreased after surgery, which is consistent with the results of a previous study [32]. Hausdorff et al. reported that stride time variability significantly correlated with quadriceps strength and gait speed in community-living older adults [17]. Kang et al. also found that the great stride time variability was associated with decreased leg strength and ROM in healthy older adults [33]. Taken together, in this study, stride time variability was expected to increase with the decrease in physical function after surgery; however, no significant change was observed between pre- and postoperative assessments. This result indicated that patients following TKA surgery could walk at hospital discharge as stably as preoperatively regardless of the decrease in physical function, including knee ROM, quadriceps strength, and gait speed after surgery.

The role of rehabilitation after TKA is to help patients achieve readiness for discharge to their homes by restoring independence in ambulation and transfers, joint movement, and muscle strength [34, 35]. Early initiation of rehabilitation treatment after surgery is important for gaining the maximum benefit from TKA [3–5] and is useful for improving gait and balance ability after surgery [5, 36]. In addition to the previous studies, the results in the present study indicated that the early initiation of rehabilitation treatment may be beneficial for acquiring gait stability at hospital discharge that is comparable to the preoperative gait stability. Further study should assess the effect of early initiation of rehabilitation on gait stability at hospital discharge.

This study has several limitations. First, the study group was a convenience sample, and the small sample size may have affected the results of this study. Second, 23 patients needed a cane during walking at hospital discharge; therefore, we could not exclude its influence on gait fluctuation. Third, we did not measure balance ability, mental status, and executive function. Previous studies reported that stride time variability correlated not only with physical function including balance ability [16] but also with mental status such as confidence in their ability to perform activities without falling [9] and executive function [37]. Future research should examine the effect of balance ability, mental status, and executive function on stride time variability at discharge. Finally, we did not investigate whether falls actually occurred after discharge. Further study is needed to investigate whether falls actually occurred over a long period after discharge.

## Conclusions

This study confirmed that stride time variability remained unchanged between pre- and postoperatively assessments in a cohort of patients with primary unilateral TKA. The present study findings indicated that patients following TKA surgery could walk at hospital discharge as stably as preoperatively regardless of the decrease in physical function, including knee ROM, quadriceps strength, and gait speed after surgery.
